# Universal Scaling for the Exit Dynamics of Block Copolymers from Micelles at Short and Long Time Scales

**DOI:** 10.1021/acs.macromol.1c02387

**Published:** 2022-01-24

**Authors:** Maria S. Pantelidou, Fabián A. García Daza, Josep Bonet Avalos, Allan D. Mackie

**Affiliations:** † Departament d’Enginyeria Química, ETSEQ, Universitat Rovira i Virgili, Tarragona 43007, Spain; ‡ Department of Chemical Engineering, The University of Manchester, Manchester M13 9PL, United Kingdom

## Abstract

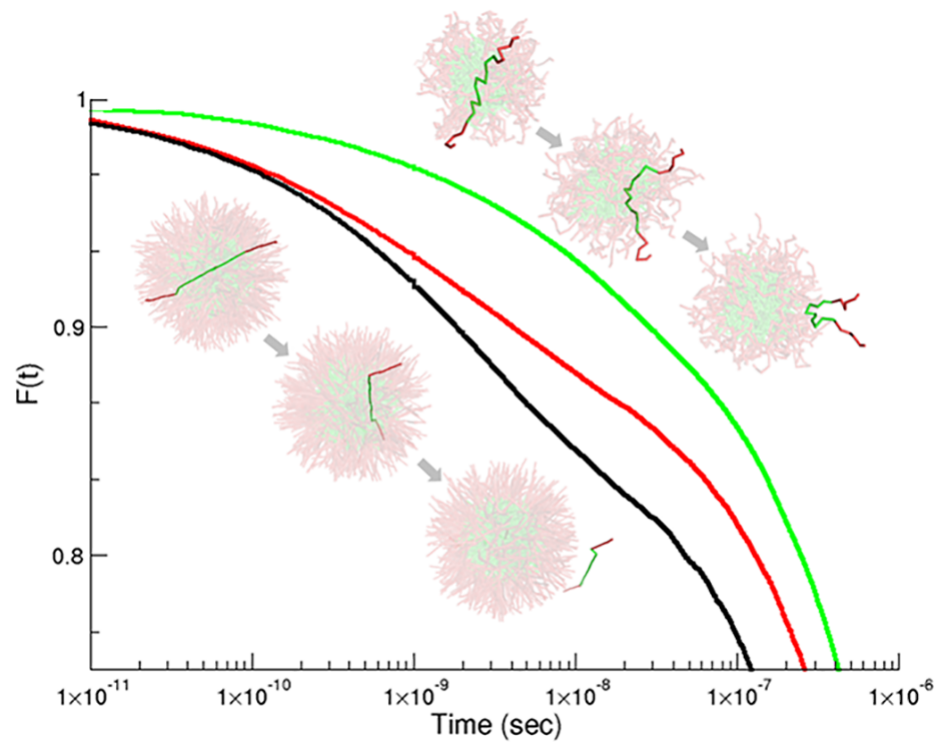

The correlation function for the exit of poloxamer copolymers from equilibrated micelles is found to show up to four regimes depending on the chain flexibility: an initial fast reorganization, a logarithmic intermediate regime, followed by an exponential intermediate regime, and a final exponential decay. The logarithmic intermediate regime has been observed experimentally and attributed to the polydispersity of the polymer samples. However, we present dynamic single-chain mean-field theory simulations with chains of variable flexibility which show the same logarithmic relaxation but with strictly monodisperse systems. In agreement with our previous studies, we propose that this logarithmic response arises from a degeneracy of energy states of the hydrophobic block in the micelle core. For this to occur, a sufficiently large number of degenerate conformational states are required, which depend on the polymer flexibility and therefore should not be present for rigid polymers. Experimental results for monodisperse polymeric samples claiming the absence of such a logarithmic response may also lack a sufficient number of hydrophobic blocks for the required number of configurational states for this type of response to be seen. The insight gained from analyzing the simulation results allows us to propose a modified Eyring equation capable of reproducing the observed dynamic behavior. On scaling experimental results from different sources and systems according to this equation, we find a unique master curve showing a universal nature of the intermediate regimes: the logarithmic regime together with the secondary exponential decay. The terminal exponential regime at long times proposed by the standard Halperin and Alexander model is beyond the range of the data analyzed in this article. The universality observed suggests an entropic origin of the short-time dynamic response of this class of systems rather than the polydispersity.

## Introduction

Poloxamers are triblock copolymers with a central hydrophobic polyoxypropylene block (PPO) between two external hydrophilic polyoxyethylene blocks (PEO). These copolymers are also available commercially under the trade name of Pluronics. Because of their amphiphilic nature, when they are surrounded by solvent molecules above a specific concentration called the CMC, they can self-assemble into aggregates known as micelles, driven by the hydrophobic effect.^1,2^ Even after reaching thermal equilibrium, the micelles are constantly evolving in shape and composition due to the low aggregation free energy involved in the hydrophobic effect. In the standard interpretation of this equilibrium, two different dynamic processes are proposed. The first is a fast process characterized by the insertion and expulsion of single surfactant chains from the micelles.^3^ On the other hand, the second is much slower and is characterized by the fusion and fission of the micelles..^4−10^ Typically, there is a difference of ∼6 orders of magnitude between the time scales involved in these two processes.^11,12^ Micelle relaxation is of particular importance because it controls micellar stability, which is important in many applications involving processes such as foaming, emulsification, detergency, wetting, and solubilization.^13^ Consequently, a detailed understanding of the associated molecular mechanism is expected to contribute key insights into micelle behavior as well as to serve as a basis for future experimental studies and applications.

Over the past few decades, numerous experimental and theoretical studies have focused on both dynamical processes.^3,14,15^ However, in this work we are particularly interested in the faster process where individual chains are exchanged with the bulk solution. In this case, the most established theory is the one developed by Aniansson and Wall,^4,5,16^ whose stepwise model assumes that for short surfactants the exchange between the bulk solution and micellar aggregates undergoes a single-exponential decay. Later, Halperin and Alexander^3^ adapted the same model, but with diblock copolymers as surfactants in a solvent of low molecular weight. They also proposed a single-exponential decay in the relaxation curves. Subsequently, much research on micelle kinetics has been done by using different experimental techniques such as temperature jump,^3^ fluorescence,^17^ ultrasonic absorption spectrometry^18^ and time-resolved small-angle neutron (TR-SANS).^19−21^ In these experiments a single-exponential decay in the relaxation process was observed, which allegedly confirmed the validity of these models for micelle dynamics.

However, more recent experimental works have found a much richer behavior, including a broad logarithmic time dependence at shorter times instead of the expected exponential decay.^20,22^ In an attempt to explain this logarithmic relaxation, Lund and co-workers^20,22,23^ ascribed this behavior to the polydispersity of the triblock copolymers. In their model, surfactants have to overcome an energetic barrier to leave the micelle, and so copolymers with different chain lengths are expected to face different barrier heights. However, it is not clearly explained why this should give rise specifically to a logarithmic decay, since the distribution of chain lengths due to polydispersity is somehow arbitrary. Simulation methods such as Monte Carlo (MC),^24^ molecular dynamics,^14,15^ and dissipative particle dynamics^25−27^ have been used to provide additional insight into the dynamics of micelles. In particular, a dynamic single-chain mean-field (SCMF) theory has been used to simulate the behavior of poly(ethylene oxide)–poly(propylene oxide)–poly(ethylene oxide) triblock copolymer systems.^28,29^ The dynamic SCMF has similarities to other dynamic mean-field density functional theories found in the literature.^30^ However, SCMF uses explicit nonoverlapping configurations instead of Gaussian statistics for the chain conformations, which significantly changes the resulting equilibrium and dynamic behavior.^31,32^ Despite using a strictly monodisperse distribution of chain lengths in the simulations, we found a similar logarithmic decay to the experiments. This suggests that the observed behavior must be caused by an inherent physical property of polymeric micelles and not to a particular polydispersity in the size distribution of the samples. The observed logarithmic decay was speculated to arise from a degeneracy of energy states related to the conformational space of the hydrophobic block of the copolymer in the core. This degeneracy is broken on exiting the micelle, giving rise to an effective energetic barrier distribution. Although this hypothesis is valid for the chains escaping at short times, at longer times the exit time of the copolymer is of the same order of magnitude as the diffusion in conformational space, and therefore, it cannot be expected for the chain to be expelled from the micelle without a change in conformation, as required by the hypothesis. Hence, the observed nonexponential decay must be related to the coupling of at least two different dynamic processes rather than to merely an energy barrier distribution. The view that we develop in this article is based on the following two points: (a) As indicated in our previous work,^28,29^ different chain conformations experience different barrier heights to exit the micelle; therefore, hairpin conformations of the triblock copolymer are more likely to exit the micelle than more extended conformations. (b) There is an entropic barrier to reach such a hairpin shape, whose height depends on the initial chain conformation.

These hairpin conformations are depleted during the initial stages, and other chains have to diffuse through the entropic barrier in conformational space until they can become ready to exit the micelle. Such a diffusive process introduces additional dynamics, which does not depend on the height of the exit energetic barrier. Although the hypothesis is oversimplified, it contains the main ingredients needed to explain the different regimes observed experimentally. In addition, this hypothesis allows the relevance of such an intermediate nonexponential decay to be related to the size of the chain conformational space. For example, stiffer triblock copolymers with longer Kuhn segments have their conformational diffusion suppressed, and their behavior should be dominated by a purely exponential decay. On the other hand, more flexible chains of the same length should display a broad intermediate nonexponential behavior. The simulations conducted in this article show that the breadth of the nonexponential decay is directly related to such a chain property.

Interestingly, a recent article^33^ has further explored the consistency of literature models based on polydispersity to explain their experimental results. The authors used Förster resonance energy transfer (FRET) to study the exchange dynamics of complex coacervate core micelles and found that the literature models required energies below the one expected for their system and speculate on the need for additional factors besides polydispersity to properly describe the observed experimental dynamics. In a further twist to their discussion, they noted that in experiments with monodisperse core blocks no logarithmic relaxation had been observed but rather a single-exponential time decay in agreement with the standard Halperin and Alexander model. At first sight, these monodisperse experimental results might appear to contradict the conclusions of the SCMF dynamic simulations already commented on.^28,29^ However, it should be noted that these experimental works use only poly(ethylene oxide) polymers (PEO) with *n*-alkyl ethers: C_*n*_–EO_5_ and C_*n*_–EO_10_–C_*n*_, where *n* = 18, 22, 24, 28, and 30. Given that the Kuhn segment length for polyethylene is about four ethylene monomers (C_2_H_4_),^34^ this indicates that the hydrophobic block of these polymers have a very reduced conformational space since they range from about only two to almost four Kuhn segments. To have a distribution of degenerate energy states of the hydrophobic block in the core, it is reasonable to assume that a sufficiently large number of Kuhn segments would be needed in the hydrophobic block. The question thus arises if such a low number of segments is enough to display the required degeneracy. On the contrary, a low number of hydrophobic segments could potentially cause the observed single-exponential time decay rather than a lack of polydispersity, as suggested by the authors of these works. The question about whether the size of the conformational space, with conformations separated by entropic barriers, affects the existence of the intermediate logarithmic regime is the main subject of this article.

To help shed some light on this matter, in this study we have performed several simulations using a dynamic SCMF simulation method. In particular, we have chosen to investigate the behavior of the L44 triblock copolymer EO_10_PO_23_EO_10_ in water at 37 °C. To explore the effect of the number of Kuhn segments on the relaxation dynamics of single chains, we have artificially changed the flexibility of the L44 chains. Three cases are considered: the standard experimental chain flexibility, a more rigid chain, and a more flexible chain. We first study the equilibrium behavior for the formation of micelles as a function of their flexibility and then model the chain dynamics to observe if the flexibility can cause the logarithmic decay to disappear as is found in the experiments. In addition, we follow the conformational behavior of the chains as they leave the micelle to check for any shrinking or swelling of the chains.

Last but not least, we propose a phenomenological equation to describe the evolution of the population of tagged surfactants inside the micelle. This phenomenological equation is a modified Eyring equation with three parameters, which are related to the kinetic constant for the intermediate logarithmic regime, the kinetic constant of the ultimate exponential regime, and a crossover surfactant number. The simulation as well as the available experimental data are fitted to this equation. As a result, we obtain a collapse of all data to a master curve, which reinforces the idea of the universality of the observed behavior.

The article is organized as follows: In the next section a brief description of the simulation methodology is given as well as the main features of the coarse-grain model. In the Results and Discussion section, the simulation results are presented for the chosen L44 Pluronic surfactant with varying Kuhn lengths that allow us to model the same molecule with different chain flexibilities. An analysis of these results allows us to follow changes in the chain configurations on exiting the micelle and propose a modified Eyring equation that shows that all the experimental data follows the same master equation. We finish the article in the Conclusions section with a summary of the most important findings.

## Simulation Methodology and Model Details

The simulation methodology used in this work has already been fully described in previous publications,^35−38^ and so in this section only the most important details are included. First of all, a description of the single-chain mean-field theory is given as well as the dynamic version of the same theory. This is followed by an introduction of the coarse-grain model that was developed for the L44 triblock copolymer^38^ and the manner in which we have introduced the different chain flexibilities into the polymer.^39^

### Single-Chain Mean-Field (SCMF)Theory

**Figure 1:**
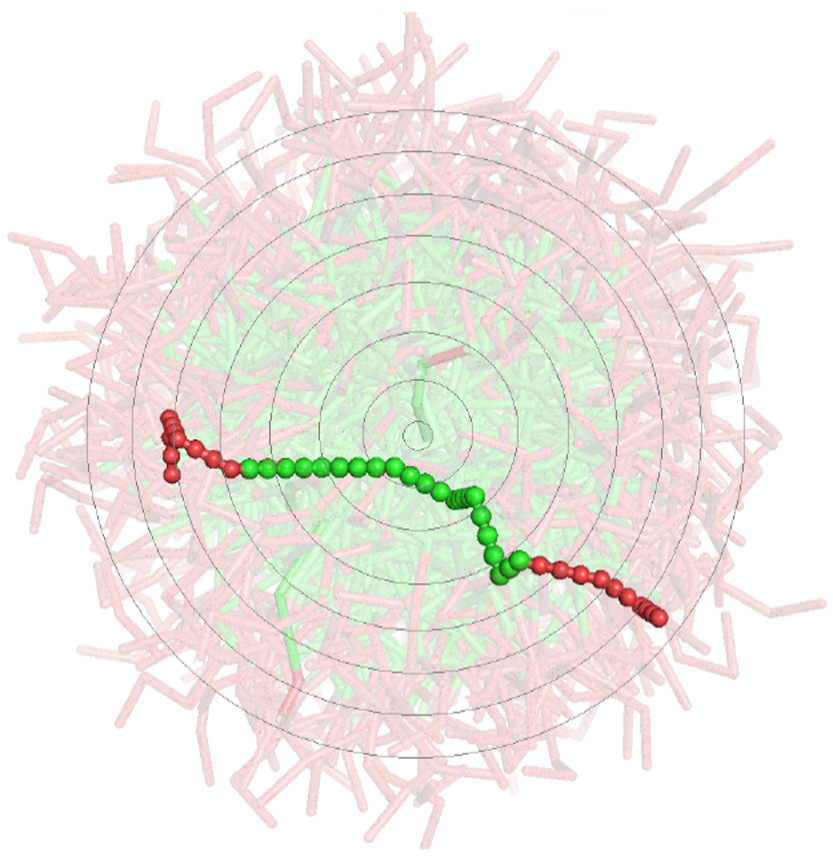
Schematic diagram of a specific surfactant chain interacting with the mean molecular fields of a micelle where the EO monomers are shaded red and the more hydrophobic PO monomers green. The circles indicate the one-dimensional discretization of the molecular densities used in this work.

The main idea behind the SCMF theory is to simulate a specific chain that interacts with the other surrounding molecules by way of mean molecular fields (see Figure 1). Consequently, a representative set of conformations {α} of this central chain needs to be sampled to self-consistently generate the statistical weight of each chain conformation α, from which the average fields are calculated. A given chain conformation is expressed as the collection of positions of all its monomers, , where  is the number of monomers of the chain. Finally, **r**_*i*_[α] stands for the position of the *i*th monomer in the configuration α.

The intramolecular interactions of the central chain, *U*_intra_(α), are calculated in an exact way whereas the intermolecular interactions with the solvent and other surfactants, *U*_inter_(α), are calculated within a mean-field approximation. The exact evaluation of the intrachain interactions allows us to keep track of the self-avoidance and conformational restrictions, which are very important for the kinetic problem that we address. The mean molecular fields are calculated by minimizing the system free energy  in a self-consistent manner using the set of chain configurations. This yields the probabilities of the different configurations, *P*[α], and any other property of interest.

The free energy functional is analogous to a density functional theory in conformational space and is expressed as follows:
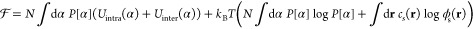
1where *N* is the number of chains in the simulated system, *k*_B_ is Boltzmann’s constant, *T* is the temperature, ϕ_s_(**r**) is the solvent volume fraction, *c*_s_(**r**) is the solvent number concentration as a function of the position **r**, and . The first term in this equation is the energy of the system, whereas the second and third terms account for the configurational and translational entropies of the chains and solvent, respectively. The solvent volume fraction is given as ϕ_s_(**r**) = *c*_s_(**r**)*v*_s_ where *v*_s_ is the volume of a solvent molecule.

The free energy in eq 1 is minimized subject to the incompressibility condition, which imposes that the available volume is completely filled by the solvent and surfactant molecules at any position **r**
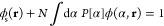
2where ϕ(α,**r**) is the volume fraction of chain conformation α at a position **r**, which is given by , where *v*_*p*_ and *c*_*p*_ refer to the volume and concentration of the chain monomers.

The volume-filling constraint in eq 2, which accounts for the repulsive short-range forces, is introduced in the minimization of the free energy via a Lagrange multiplier field π(**r**). After evaluating  and , we obtain the corresponding chain probabilities, *P*[α], and the solvent number concentration, *c*_s_(**r**), as follows:
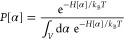
3
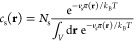
4where *V* is the volume of the system and *N*_s_ is the total number of solvent molecules. *H*[α] is the SCMF Hamiltonian, which contains the total intramolecular and intermolecular interactions of conformation α with the surrounding fields. An analytical form of this Hamiltonian reads
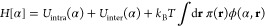
5A more detailed expression of the Hamiltonian for the Pluronic surfactants in water is given later in the section on model details. In this work we are only interested in the behavior of spherical micelles, and so the mean fields were discretized into concentric spherical layers starting from the center of the simulation cell. A schematic diagram of these spherical shells is given in Figure 1.

### Dynamic SCMF Scheme

To study the relaxation of the surfactants in the micelle, we use a dynamic version of the SCMF scheme which has already been applied in previous studies.^28,29^ This scheme is based on the generation of local displacements of an initial ensemble of sampling chains, {α_0_}, within the already generated mean fields. New chain configurations are generated independently of each other, in agreement with our mean-field approach, by using a random movement in the spirit of dynamic Monte Carlo. Each new conformation is then accepted or rejected with a given probability, following the Metropolis algorithm eq 6, according to the change in the energy between the new and old conformations, α_*n*_ and α_0_, respectively. In this way, detailed balance is maintained and the correct sampling of the equilibrium canonical distribution for chain conformations is achieved. The probability of acceptance can be written as
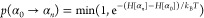
6In every cycle a new set of conformations, {α_*n*_}, is generated, after which the mean-field concentration fields are updated by solving the SCMF equations. This cycle is equivalent to a step forward in time, Δ*t*, the size of which needs to be determined independently to relate the simulation cycles to a physical time scale.

### Model Details

**Figure 2:**
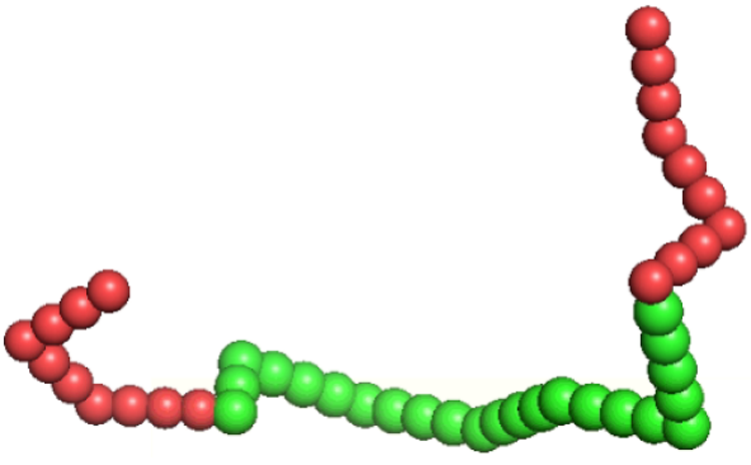
Typical chain configuration of the L44 triblock copolymer (EO_10_PO_23_EO_10_) with a semiflexible chain where the hydrophilic EO monomers are shaded red and the hydrophobic PO monomers green.

In this work we implement a coarse-grain model for the triblock copolymer surfactant L44 Pluronic^38^ (EO_10_PO_23_EO_10_) which has a relatively low molecular weight of 2200. L44 is a linear chain for which we choose to model both the EO and PO monomers as beads of the same diameter σ. Figure 2 depicts a typical chain configuration where the green spheres correspond to the hydrophobic propylene oxide monomers (PO, CH(CH_3_)CH_2_O) and the red spheres correspond to the hydrophilic ethylene oxide head monomers (EO, CH_2_CH_2_O). The distance between consecutive beads is chosen to be equal to σ, while the monomer interactions are modeled by square well potentials at the center of each bead with inner radius σ, outer radius 1.62σ, and a well depth which depends on the particular interaction that is to be modeled. The chain flexibility is taken into account by using Kuhn segments, where a chosen number of monomers are taken to form a rigid block although we allow complete flexibility between consecutive blocks. A larger number of rigid monomers embedded in each Kuhn segment lead to a more rigid chain with a smaller conformational space.

The details of the SCMF Hamiltonian already given in eq 5 depends on the model being considered. In this particular case, the Hamiltonian includes the interactions between the EO, PO, and water solvent molecules, together with the steric interactions. Hence, the Hamiltonian takes the following form:
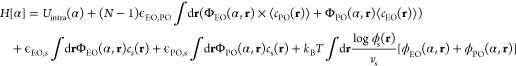
7where the first term corresponds to the intramolecular interactions; the second, third, and fourth terms are the intermolecular interactions between EO–PO, EO–solvent, and PO–solvent molecules, respectively, together with their corresponding average concentration fields ⟨*c*_EO_(**r**)⟩ = ∫dα *P*[α]*c*_EO_(α,**r**), ⟨*c*_PO_(**r**)⟩ = ∫dα *P*[α]*c*_PO_(α,**r**), *c*_s_(**r**), and their available interaction volumes Φ_EO_(α,**r**) and Φ_PO_(α,**r**). Finally, the last term represents the steric interactions, arising from the hard-core repulsion (or excluded volume) of the different molecules, expressed here as an incompressibility condition. The values of the interaction energy parameters were taken from our previous work,^38^ where an optimization procedure was performed to reproduce the available experimental CMC literature values. The values are ϵ_EO,PO_ = 0.006*k*_B_*T*/*z*, ϵ_EO,s_ = 0.5*k*_B_*T*/*z*, and ϵ_PO,s_ = 2.1*k*_B_*T*/*z*, with *z* = 26 as the coordination number. These values are closely related to the equivalent Flory–Huggins parameters. The coarse-grain nature of our model prohibits its applicability over a range of temperatures and is limited to the temperature of 37 °C of the experiments to which the parameters were optimized.

The stochastic character of our dynamic SCMF has no physical time scale as such. Consequently, for the purpose of comparing with experimental data, we estimate the physical time scale of our simulations, *t*. To this end, we compare the diffusion of the free chains in the simulations, *D*_SCMF_, to the one expected experimentally, *D*. The diffusion of a free chain in our simulation can be estimated in terms of the MC cycles, *t*_cyc_
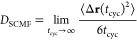
8where  is the average displacement of the center of mass of the *N*_{α}_ chains in the set {α}. Furthermore, we used the following dimensionless equation to convert the MC cycles into experimental time *t*.
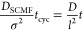
9The diffusion constant *D*_SCMF_ is given in units of σ^2^/cycle, whereas *l* is the physical dimension of the monomers. In our case the monomers have a diameter σ of ∼0.2 nm which followed from a comparison of available experimental data and the diffusion constant calculated from the Stokes–Einstein expression
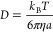
10where the viscosity of the solvent (water) was taken to be η = 6.91 × 10^–4^ kg m^–1^ s^–1^ and the hydrodynamic radius *a* is taken from the Rouse model, where .

## Results and Discussion

In this work we aim to study the effect of chain flexibility on the dynamic exchange of surfactants between micelles and the bulk solution. To this end, we studied the behavior of the L44 triblock copolymer in water at 37 °C by using SCMF calculations. This particular block copolymer was selected as it is a relatively short Pluronic (EO_10_PO_23_EO_10_) but still long enough to allow for different flexibilities to be introduced. Three degrees of surfactant chain flexibility have been chosen. The different flexibilities are obtained by adjusting the stiffness of the surfactant chain through the number of monomers in each Kuhn segment in the hydrophilic head *l*_*k*_^H^ and the hydrophobic tail *l*_*k*_^T^ blocks (see Table 1). Only in the semiflexible case does the surfactant correspond to a realistic experimental system, namely the L44 Pluronic surfactant. In the other two cases, flexible and rigid, the resulting chain has not been chosen to represent any given known experimental system.

**Table 1:** Equilibrium Properties of L44 Triblock Copolymers (EO_10_PO_23_EO_10_) in Water at 37 °C from SCMF Calculations: *l*_*k*_^EO^ and *l*_*k*_^PO^ Are the Size of the Kuhn Segments of the Hydrophilic (EO) and Hydrophobic (PO) Blocks, Aggregation Number (*N*), Minimum Chemical Potential Differences, and Critical Micelle Concentration

case study	*l*_*k*_^EO^(σ)	*l*_*k*_^PO^(σ)	*N*	min((μ_*N*_^0^ – μ_1_^0^)/*k*_B_*T*)	CMC (mol/L)
flexible	2	2	91	–9.2	5.1 × 10^–3^
semiflexible	3	4	145	–9.4	4.0 × 10^–3^
rigid	10	20	320	–9.7	3.3 × 10^–3^
					

**Figure 3:**
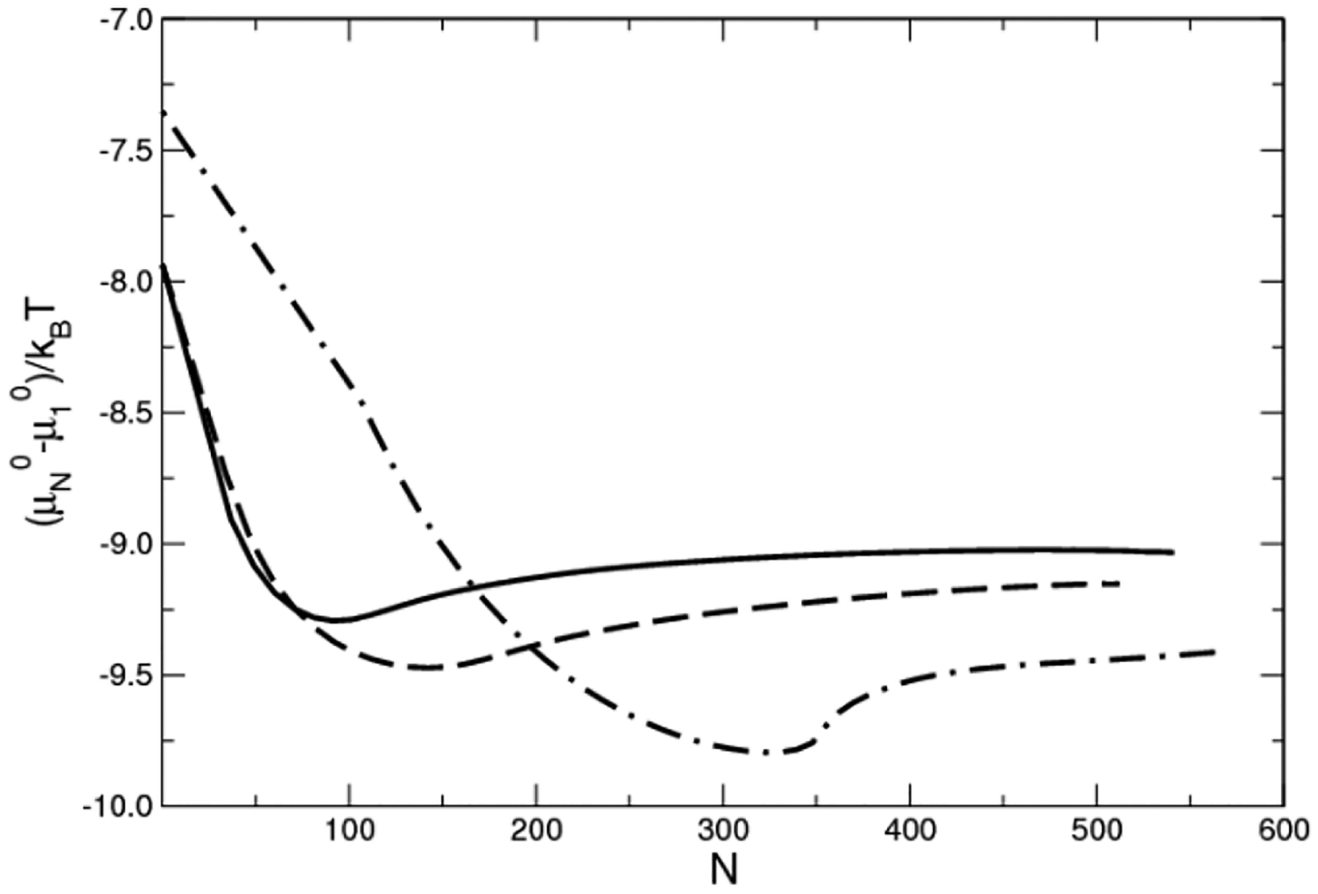
Standard chemical potential difference, (μ_*N*_^0^ – μ_1_^0^)/*k*_B_*T*, versus the aggregation number, *N*. Solid, dashed, and dot-dashed lines indicate the flexible, semiflexible, and rigid cases, respectively.

Before we can study the dynamics of our systems, we need to estimate their equilibrium properties. On applying the equilibrium SCMF simulation technique already described, it is possible to calculate the standard chemical potentials of chains in micelles containing *N* chains, μ_*N*_^0^, as compared to those in the bulk solution, μ_1_^0^.^40^Figure 3 depicts the resulting standard chemical potential difference (μ_*N*_^0^ – μ_1_^0^)/*k*_B_*T* as a function of the number of surfactants in the micelle. In all three cases the systems prefer to self-assemble into micelles rather than form a homogeneous solution, as evidenced by the appearance of a minimum value in our SCMF results where the preferred micelles are expected to appear. Further details of these calculations can be found in our previous work.^38,39^ In Table 1, the minimum of the standard chemical potential difference, as a function of the aggregation number *N*, is given together with the corresponding aggregation number and CMC estimated by log CMC = min((μ_*N*_^0^ – μ_1_^0^)/*k*_B_*T*).

**Figure 4:**
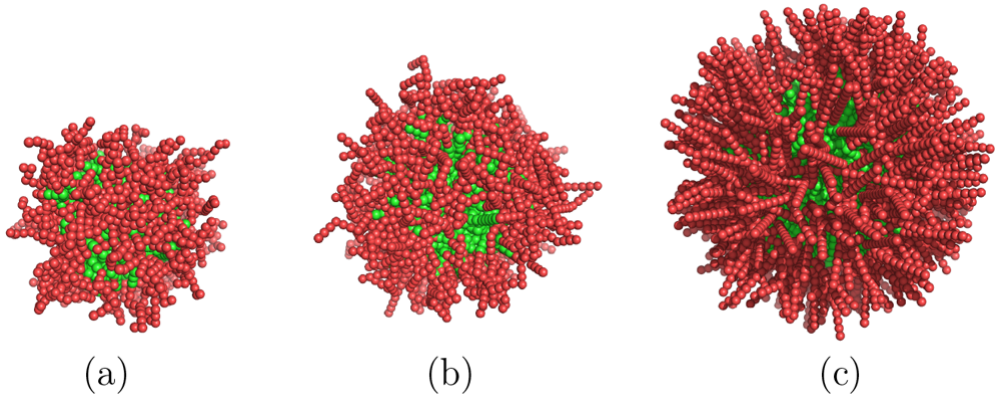
Schematic diagram of typical micelle configurations for the three case studies: (a) flexible, *N* = 91; (b) semiflexible, *N* = 145; and (c) rigid, *N* = 320. The EO monomers are shaded red and the more hydrophobic PO monomers green.

As expected, the aggregation number of the minimum free energy of the micelles depends on the flexibility of the chain, with larger micelles being preferred for stiffer chains. In particular, for the flexible surfactant the preferred micelle has an aggregation number of 91, the semiflexible surfactant is significantly larger containing 145 chains, and the rigid case is much larger, with 320 surfactants. In Figure 4 schematic representations of the equilibrium micelles are given for the three different case studies of chain flexibilities. These diagrams plot the most probable conformations of the chains corresponding to the free energy minimum for each case study.

For the dynamic simulations, we consequently decided to use 91 surfactants for the flexible surfactant and 145 for the semiflexible case, in line with the preferred micelle size for these systems. However, we chose an aggregation size of 150 for the rigid surfactant, instead of the preferred micelle size, to reduce the computational time needed for a system with such a large micelle. This can be achieved by a suitable choice of the number of available surfactants within the simulation box. The size of the micelle is not expected to have a strong effect on the micelle dynamics.

**Figure 5:**
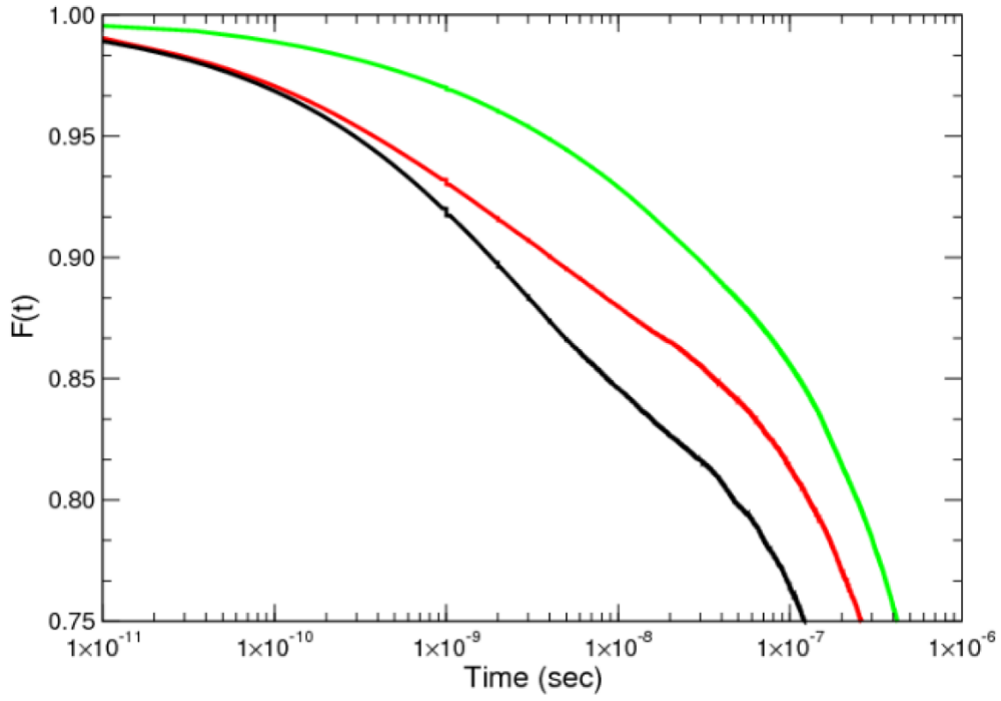
Dynamic equilibrium exchange correlation function, *F*(*t*), as a function of time for a linear–log plot for the three case studies. From left to right: flexible in black, semiflexible in red, and rigid in green.

To study the dynamic equilibrium exchange of the micelle chains with the bulk solution, we used a correlation function, *F*(*t*), similar to the one used in the experimental TR-SANS studies,^22,41,42^ namely
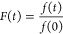
11The chains that are inside the micelle are labeled at time *t* = 0 and keep this label throughout the simulation. This leads to the initial number of labeled chains inside the micelle, *f*(0). During the simulation, the number of labeled chains inside the micelle, *f*(*t*), is tracked as a function of time. The micelle is in thermal equilibrium during the whole process; namely, no variation in the average fields is produced due to the fact that new chains from the bulk replace the ones which exit the micelle, but the former may not be tagged. By initially tagging the chains inside the micelle, we create a virtual nonequilibrium state which causes the subsequent mixing process between tagged and untagged chains until an eventual equilibrium between the tagged molecules in the bulk and within the micelle. Therefore, the final equilibrium distribution of tagged chains inside the micelle is not zero. The time span of our simulations is shorter than the one required to reach such a new equilibrium. The results of the evolution of the labeled surfactant chains over time for each case of flexibility can be seen in Figure 5 in a linear–log plot, which helps to highlight the presence of the logarithmic decay as a straight line.

As previously mentioned in the Model Details section, to connect our findings with experimental data, we need to convert the simulation cycles, *t*_cyc_, into physical time. This was performed by using eqs 9 and 10 and the main results of the estimated simulation diffusion coefficients. The resulting conversion of the simulation cycles into time in seconds can be found in Table 2.

**Table 2:** Diffusion Coefficient from SCMF Simulations and Physical Time for Each Chain Flexibility

case study	*D*_SCMF_ (σ^2^/cycle)	physical time, *t*_cycle_ (*t*_cyc_ s/cycle)
flexible	0.5 × 10^–3^	2.6 × 10^–13^
semiflexible	1.3 × 10^–3^	7.0 × 10^–13^
rigid	58.8 × 10^–3^	17.4 × 10^–13^

In the cases studied in this work we can detect up to three regimes depending on the chain flexibility (see Figure 5). In all there is an initial regime at short times (*t* < 10^–10^ s), which corresponds to a very fast exit of the chains that are ready to leave at *t* = 0 due to their appropriate instantaneous chain conformation. This regime may be followed by an intermediate logarithmic regime with a width of about 2 orders of magnitude (10^–10^ < *t* < 10^–8^ s). A final regime can also be identified for *t* > 10^–7^ s which shows an exponential decay. Later in this article we will discuss in more detail this final exponential relaxation behavior with respect to the Halperin and Alexander model for block copolymers and the possible existence of an intermediate exponential regime followed by a terminal exponential decay.

On inspection of Figure 5 we find that an intermediate logarithmic regime appears for the flexible and semiflexible surfactants and takes on the form of a characteristic straight section in the linear–log plots. Such a straight line section is indicative of a logarithmic decay, *F*(*t*) ∼ – log(*t*) which emerges for the cases where the surfactants are sufficiently flexible and is absent for the more rigid chain. The signature of a logarithmic decay has been observed by several authors in experiments^20,22^ as well as from our previous simulation studies.^28,29^ As already mentioned, the main thesis of the experimental groups is to attribute the appearance of this peculiar logarithmic regime to the polydispersity of the available polymer samples. However, our previous and present simulation results give a similar logarithmic behavior by using a purely monodisperse distribution of chain length. Therefore, as we have already argued,^28,29^ this logarithmic trend needs to be considered as being caused by some intrinsic property of the micellar system rather than only to the polydispersity of the samples. Polydispersity will play some role, but obviously it cannot explain the simulation results performed with strictly monodisperse samples. The present analysis thus focuses on the impact of the rigidity of the hydrophobic block in the form of such intermediate decay, which, as our analyses reveal, must be the key element to understand the apparently disparate interpretation of the experimental results. Interestingly, in the case of the rigid block copolymers, no intermediate logarithmic regime is observed. The representation of the simulated data in a log–log plot (not shown) does not change this interpretation of the data.

**Figure 6:**
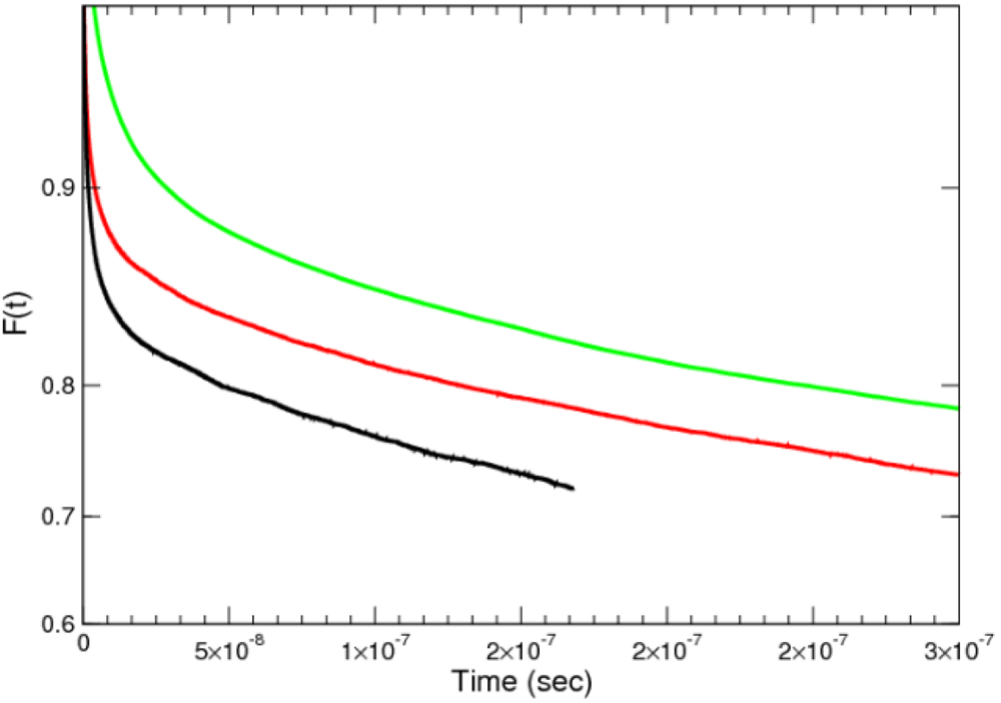
Dynamic equilibrium exchange correlation function, *F*(*t*), as a function of time using a log–linear plot for the three case studies. From left to right: flexible in black, semiflexible in red, and rigid in green.

To emphasize the form of the terminal regime, we have plotted our simulation results using a log–linear plot in Figure 6. If an exponential regime is attained, the curves should be straight lines in this plot which we expect to occur after around *t* ∼ 10^–7^ s. In the cases of the rigid and semiflexible chains it was possible to begin to enter this regime; however, in the case of the flexible chain, it was not possible to simulate sufficiently long times.

The appearance of a logarithmic decay thus requires the surfactant chains to have a sufficient degeneracy of the energy states in the micelle core together with a preferred conformation on exit, which does not exist for the most rigid chain. This degeneracy is a result of the different conformations of the hydrophobic block segment in the core and so must require a sufficient number of hydrophobic monomers. On inspection of Figure 5, we find that the extension of this logarithmic regime is significantly larger for the flexible chain and appears over almost 3 orders of magnitude. In the case of the semiflexible chains this regime is reduced to being observed over closer to 2 orders of magnitude, although it is difficult to be categoric due to the nature of the data available. What is clear is that for the rigid chains this regime is not present. Our results thus indicate that the two hydrophobic Kuhn segments of the rigid chains are not enough to produce the effect, but the six Kuhn segments of the semiflexible chains is already sufficient.

If we test this view by comparing with the experimental studies where only an exponential decay was observed, we find that only the *n*-alkyl ethers C_*n*_–PEO_5_ and C_*n*_–PEO_10_–C_*n*_, with *n* = 18, 22, 24, 28, and 30, were used, as already mentioned in the Introduction. The resulting Kuhn hydrophobic blocks of these polymers range from about two to almost four segments. According to the simulation results from this work, these surfactants may not be long enough to allow for a clear logarithmic regime and would explain why none was observed. Clearly, it would be instructive to perform experiments for monodisperse surfactants with larger hydrophobic block lengths to see if this logarithmic behavior then appears. It should be noted that the first regime, where a rapid rearrangement of the surfactant occurs due to the initial labeling of surfactants, does not appear in the experimental results.

To better understand the behavior of the block copolymers, we examined any changes in the chain conformations on leaving the micelle. In particular, we calculated the radius of gyration of the central hydrophobic block as a function of the radial distance of the center of mass of these PO blocks with respect to the center of the micelle.

**Figure 7:**
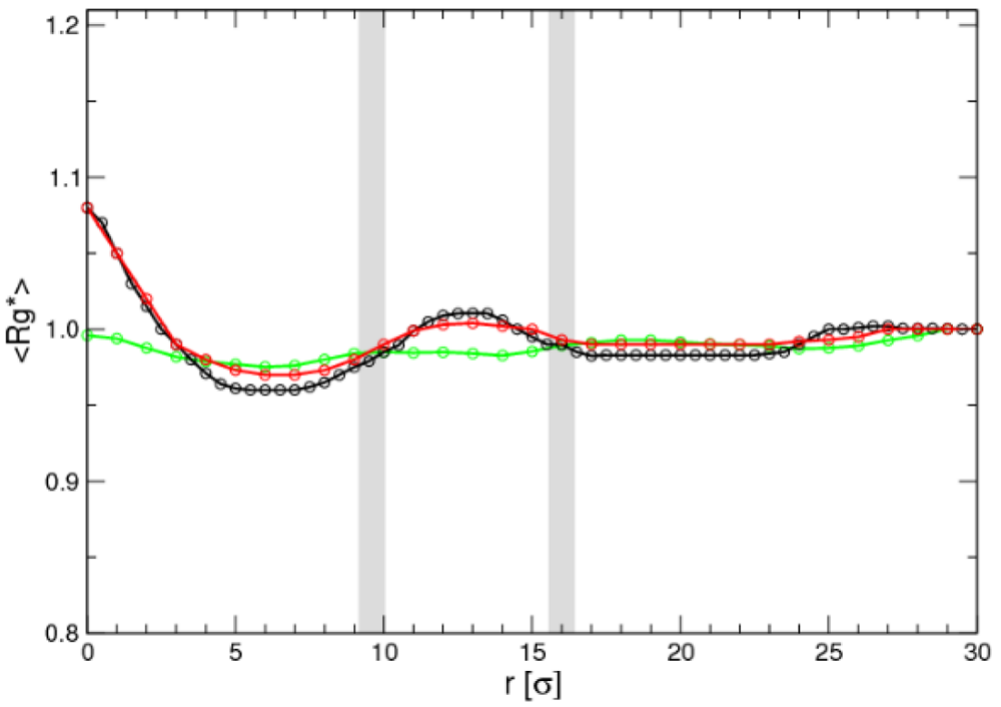
Radius of gyration of the PO block relative to the bulk solution value (*R*_g_*) with respect to the distance from the micelle center for each case study: flexible in black, semiflexible in red, and rigid in green. The blue dashed lines represent the interfaces between the core of the micelle and corona (left) and between the corona and bulk solution (right), while the thickness corresponds to the differences between the three case studies.

Specifically, according to Figure 7, there is a significant change in *R*_g_ for the flexible and semiflexible chains when moving from the center of the micelle to the threshold of the corona, while the rigid chain does not show any significant change. This fact supports the opinion that there are preferred conformations on exit for the systems that are flexible enough as to produce some sort of hairpin structure with a crumpled hydrophobic core, as this last figure suggests.

On crossing the micelle corona, all chains display a slight swelling which is much slighter than the collapse for the flexible and semiflexible chains and similar for the rigid chains. On leaving the corona, the chains attain the bulk solution value, which may mean a slight swelling or collapse depending on the chain flexibility.

**Figure 8:**
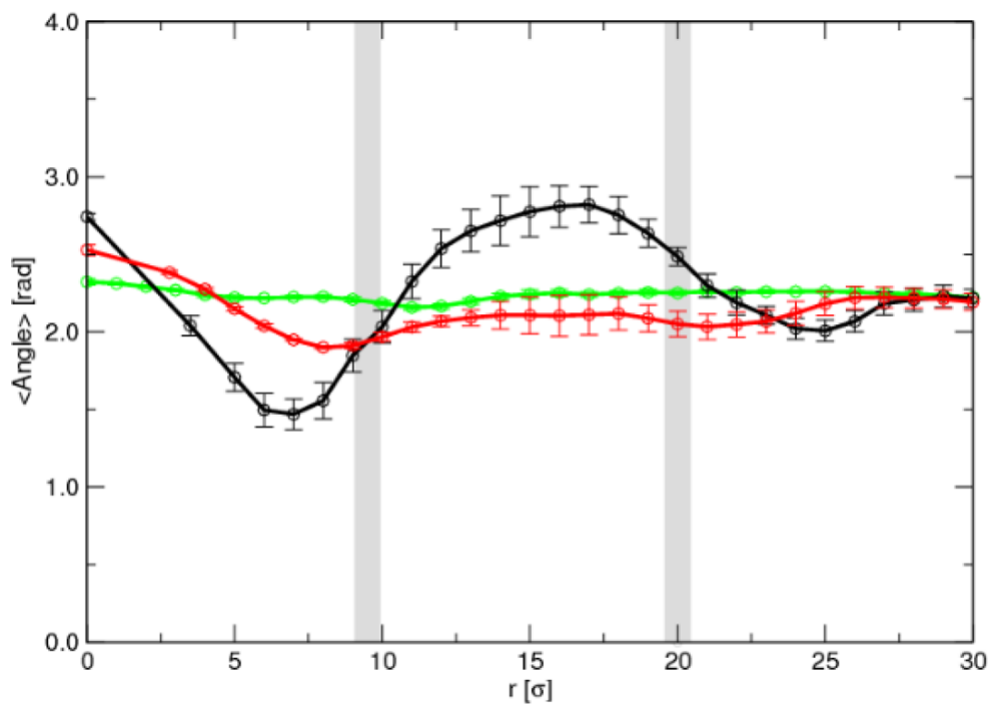
Average angle between the PO and EO blocks as a function of distance from the micelle center for each case study: flexible in black, semiflexible in red, and rigid in green. The blue dashed lines represent the interfaces between the core of the micelle and corona (left) and between the corona and bulk solution (right), while the thickness corresponds to the differences between the three case studies.

In addition, we calculated the average angle between the two hydrophilic blocks as a function of distance from the micelle center for each case of flexibility. Here we define two vectors from the center of mass of the PO block to the centers of mass of the two EO blocks and calculate the resulting angle. A completely stretched chain will have an angle of 180° and will be reduced as it approaches a hairpin conformation. As can be seen in Figure 8, in all cases the angle between the PO and EO blocks decreases as the surfactants approach the EO–PO interface and then increases until the surfactants reach the corona–water interface, where they leave the micelle and enter the bulk solution. It is also remarkable that as the flexibility of the surfactants decreases, the changes in the angle between the EO–PO blocks became smaller, which arises from the fact that the conformations are more limited as the surfactant is more rigid. Again, Figure 8 confirms that the chain flexibility favors the formation of hairpin conformations that are more likely to leave the micelle than more stretched ones. As we argued in ref (29), crumpled conformations offer less contact between the hydrophobic monomers and hydrophilic corona monomers or bulk fluid, thus minimizing the energy barrier to be overcome to escape the micelle. Therefore, chain flexibility is the crucial feature underlying the nonexponential decay in the intermediate logarithmic regime.

**Figure 9:**
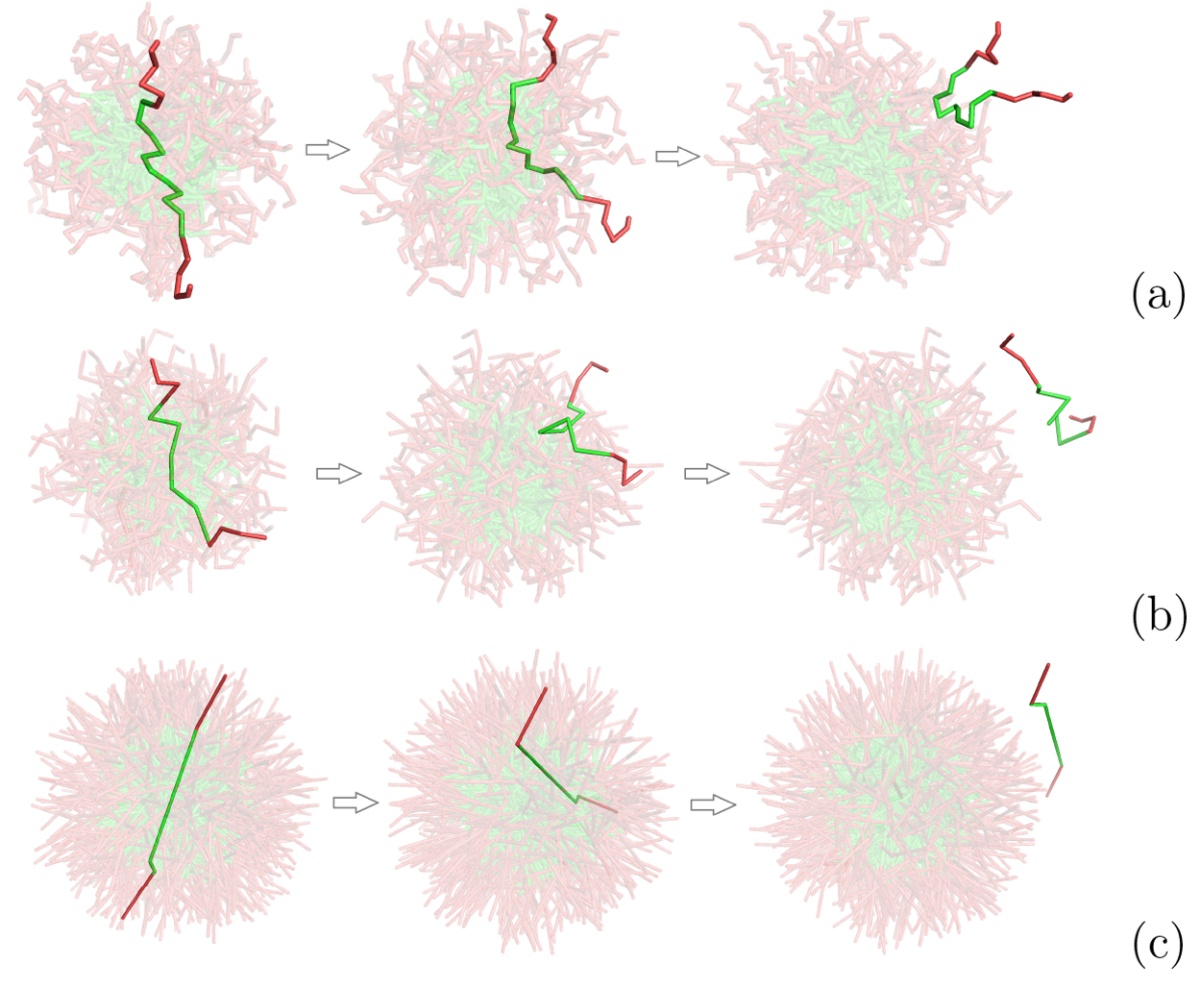
Schematic diagrams of the most likely surfactant configurations for three different distances from the micelle center (in the core, passing through the corona, and reaching the bulk) for the three case studies: (a) flexible, (b) semiflexible, and (c) rigid. The EO monomers are shaded red and the more hydrophobic PO monomers green.

TR-SANS and fluorescence spectroscopy experiments for diblock and triblock copolymer systems support the idea that the chains are either collapsed^20,23,43^ or stretched^41−44^ when leaving the micelle. However, from our calculations, we do not observe neither completely collapsed nor stretched conformations, when leaving the micelle. In Figure 9 we show a sketch of the micelle escape process for the three chain rigidities studied in this article.

To better understand the simulation results and to be able to compare with the available experimental data, we decided to try to fit the correlation function with an appropriate mathematical expression able to reproduce the main features of the dynamic process. The Eyring equation was a natural starting point since it already provides a logarithmic dependence; however, because the final regime has its own kinetics, for this system we require a modified version of the Eyring equation that includes a third parameter, namely
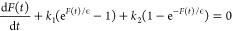
12where *k*_1_ represents the characteristic kinetic constant for the initial and intermediate regimes, *k*_2_ is indicative of the kinetic constant in the final exponential regime, and ϵ is a crossover value of the correlation function. These constants need to be fitted by using either the simulation data from the correlation function, *F*(*t*), or experimental data for the same quantity. Note that this modified Eyring equation has an additional third term with a constant *k*_2_ which gives three different regimes, as required. This differential equation can be analytically solved, yielding
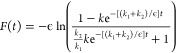
13where
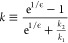
14However, the exact closed form does not reveal the subtleties of the different regimes, which are better discerned through a simple asymptotic analysis shown later on in this section. On fitting eq 12 to our correlation function simulation data for the different case studies, we find that in all cases it is possible to obtain a good fit with an accuracy of 98–99%. The fits for the three simulated cases studies, in comparison with the numerical data, are depicted in Figure S1 of the Supporting Information. In Table 3 we show the fitted parameters ϵ, *k*_1_, and *k*_2_ of the modified Eyring equation (eq 13) used in this study. As can be observed, ϵ is approximately constant with a value of about ∼2 × 10^–2^, as is *k*_2_, which has values in the range (4–7) × 10^5^ s^–1^. However, *k*_1_ changes several orders of magnitude between 10^–8^ and 10^–14^ s^–1^ on going from the flexible to the rigid chain.

**Table 3:** Results from Fitting the Modified Eyring Equation to the Correlation Function for Each Chain Flexibility

case study	ϵ	*k*_1_(*s*^–1^)	*k*_2_(*s*^–1^)
flexible	2.6 × 10^–2^	1.0 × 10^–8^	6.8 × 10^5^
semiflexible	2.0 × 10^–2^	1.0 × 10^–13^	4.1 × 10^5^
rigid	1.9 × 10^–2^	1.0 × 10^–14^	4.3 × 10^5^

Because several experimental results of correlation functions for block copolymers are available in the literature, we decided to also fit the parameters of the modified Eyring equation using the experimental data. Table 4 summarizes the estimated parameters associated with fitting eq 13 with previous experimental data.^22,41,42^ We found that in all cases the accuracy was good, about 95–99%. Plots of the fits for all the experimental data sets are available in Figures S2–S4.

**Table 4:** Results from Fitting the Modified Eyring Equation to the Correlation Function from Previous Studies Based on TR-SANS Experiments

set 1^a^	ϵ	*k*_1_ (s^–1^)	*k*_2_ (s^–1^)
PEP1–PEO20 47 °C	7.2 × 10^–2^	4.1 × 10^–10^	2.4 × 10^–9^
PEP1–PEO20 55 °C	7.9 × 10^–2^	3.6 × 10^–9^	9.4 × 10^–10^
PEP1–PEO20 60 °C	8.2 × 10^–2^	1.2 × 10^–8^	4.8 × 10^–10^
PEP1–PEO20 65 °C	10.1 × 10^–2^	1.2 × 10^–7^	4.4 × 10^–10^

set 2^b^	ϵ	*k*_1_(*s*^–1^)	*k*_2_(*s*^–1^)
1 vol % PEP–PS–PEP	8.2 × 10^–2^	1.3 × 10^–2^	0.7
6 vol % PEP–PS–PEP	8.4 × 10^–2^	3.4 × 10^–3^	3.3 × 10^–4^

set 3^c^	ϵ	*k*_1_ (s^–1^)	*k*_2_ (s^–1^)
PS–PEP-1	9.1 × 10^–2^	1.8 × 10^–5^	3.8 × 10^–4^
PS–PEP-2	9.4 × 10^–2^	7.2 × 10^–9^	4.3 × 10^–8^

a Lund et al.^22^ for poly(ethylene propylene)–poly(ethylene oxide) (PEP_1_–PEO_20_ the numbers represent the molecular weight in kg/mol) diblock copolymers.

b Lu et al.^41^ for poly(styrene)–poly(ethylene propylene)–poly(styrene) (PEP–PS–PEP).

c Choi et al.^42^ for poly(styrene)–poly(ethylene propylene) (PS–PEP) diblock copolymers with different hydrophobic (PS) lengths (⟨*N*_PS_⟩ = 255 for PS–PEP-1 and ⟨*N*_PS_⟩ = 412 for PS–PEP-2).

The experimental data implemented for the fitting were based on TR-SANS experiments. Specifically, set 1 are from the equilibrium exchange kinetics of starlike poly(ethylene propylene)–poly(ethylene oxide) (PEP_1_–PEO_20_) micelles in a 25% dimethylformamide (DMF)–water solvent mixture, at ϕ = 1*%*, for different temperatures (47, 55, 60, and 65 °C).^22^ Set 2 are from the kinetic analysis of poly(ethylene propylene)–poly(styrene)–poly(ethylene propylene) (PEP–PS–PEP) triblock micelles at two different concentrations (1 vol % PEP–PS–PEP and 6 vol % PEP–PS–PEP).^41^ Finally, set 3 are from the micelle exchange dynamics of two poly(styrene-*b*-ethylene-*alt*-propylene) copolymers, PS–PEP-1 and PS–PEP-2, in squalane with different hydrophobic (PS) repeat units, ⟨*N*_PS_⟩ = 255 and ⟨*N*_PS_⟩ = 412, respectively.^42^

According to the estimated parameters, we observe that ϵ remains approximately constant in all cases, with values of the order of magnitude of 10^–2^. However, we observe significant changes for *k*_1_ and *k*_2_. Specifically, *k*_1_ has values between 10^–10^ and 10^–5^ s^–1^, except for the cases of 1 vol % PEP–PS–PEP and 6 vol % PEP–PS–PEP in which it takes values of 10^–2^ and 10^–3^ s^–1^, respectively. Finally, with respect to the *k*_2_ parameter, we observe that it also has a wide range of values between 10^–10^ and 1 s^–1^. However, the relevant parameter for the dynamics is the ratio of these two kinetic constants, which we have denoted as γ ≡ *k*_1_/*k*_2_. Two different dynamic classes can be identified depending on whether this value is larger or smaller than 1. In particular, the simulation results belong to the class of γ < 1 where in fact γ is negligibly small (see Table 3). The following experimental results also belong to this same class: case 1 of set 1 (cf. Table 4) with a value of γ ∼ 10^–1^, case 1 of set 2 with γ ∼ 10^–2^, and the two cases of set 3, whose values are γ ∼ 10^–1^. The rest of the experimental cases correspond to the dynamic class where γ > 1, with values of γ ∼ 10–10^3^.

These experimental values are for block copolymers different from the Pluronic modeled in this paper and are in addition in different solvents and temperatures. Nevertheless, we can attempt to compare some of the main features. In particular, it is interesting to note that the ϵ parameter from our simulations has the same order of magnitude to the experiments with only small variations. In addition, the *k*_1_ parameters estimated from our simulation are also within the range of values of the experiments. Contrarily, the values of the *k*_2_ parameters are orders of magnitude higher than any of the ones from the experiments. This is probably due to a lack of sufficient data in the terminal regime with an exponential decay in the experiments, doubtlessly because the experimental block copolymers, solvents, and temperatures were chosen to highlight the intermediate logarithmic regime. Consequently, the *k*_2_ parameters, obtained on fitting to the experimental data, should be taken with caution as their lack of precision can be responsible for the appearance of these low values.

**Figure 10:**
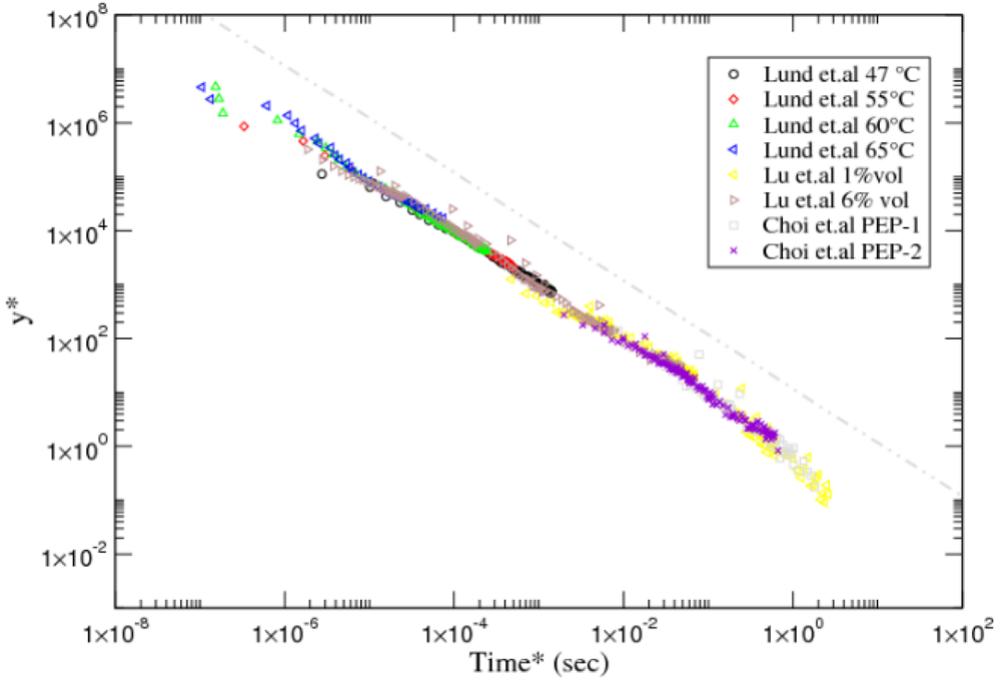
A log–log plot of the experimental data of the references given in Table 4 with the scaled variables. An additional gray dot-dashed line of slope 1 indicative of a logarithmic decay is included above the data sets. Notice that the scaling can also capture the crossover toward the exponential regime, which should appear in the region t* ≫ 1.

With the information gathered about the values of the parameters, we can return to eq 12 and analyze its properties. In the first place, let us introduce a new variable *y* ≡  exp(*F*/ϵ) and rewrite eq 12 as
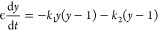
15Because *F*(0) = 1 and ϵ ∼ 10^–2^, the initial value of the variable *y* is very large. To analyze the equation in the initial stages, we introduce a rescaling of the variable, *y* = e^1/ϵ^ *ỹ*, so that *ỹ* ∼ 1. Hence, the resulting equation reads
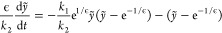
16According to this form, we can assume that *ỹ* ≫ e^–1/ϵ^. Introducing a scaling of the two variables, *t* = at* and *ỹ* = by*, eq 16 can be reduced to a parameter-free form for y* and t* by choosing
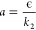
17
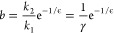
18which yields the dimensionless form
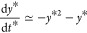
19The important aspect of this scaling of variables is that it allows us to place all the experimental data under one single master curve, which strongly supports the existence of an underlying universal behavior. Thus, taking t* as the independent variable, using the parameters given in Table 4 in eq 17 for each experimental set, and setting  as the dependent variable, we obtain the master curve given in Figure 10. This figure shows that the logarithmic behavior spans over 6 decades, involving up to eight data sets of two different groups. To make apparent the logarithmic behavior, let us consider the region in which y* ≫ 1 and, therefore
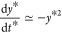
20The solution of this equation is of the form
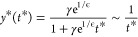
21where the last expression is valid for t* ≫ e^–1/ϵ^/γ. This asymptotic behavior can be identified as a slope of −1 in a log–log plot of y* vs t* (see the gray dot-dashed line in Figure 10). In terms of the original variables, one finds
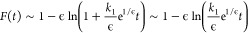
22Therefore, the logarithmic behavior is established after an initial time τ_1_ ≡ (ε/k_1_) e^−1/ε^, in the region *t* > τ_1_, where τ_1_ is also a characteristic time scale of the decay. Notice that the time scale of the logarithmic decay is governed by the kinetic constant *k*_1_ together with the crossover parameter ϵ. The crossover toward the next regime depends on whether the parameter γ is smaller or larger than 1 and is therefore system-dependent.

Let us start with the case γ > 1, which we will refer to as type *a* dynamics. In this case, the regime that appears after the logarithmic decay is determined by the point at which *ỹ* ∼ e^–1/ϵ^ > e^–1/ϵ^/γ (equivalently, y* ∼ γ > 1); namely, one enters the exponential terminal regime before the second term on the right-hand side of eq 19 becomes important. The dynamics of such a terminal regime is obtained by assuming that y* – γ is very small and therefore
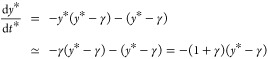
23which yields
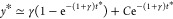
24When expressed in terms of the natural variables, one finds that, effectively, the decay is exponential:
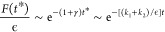
25The characteristic time scale of the exponential decay is . Thus, because 1/τ_1_ ≫ 1/τ_2_, we can asymptotically match the two solutions by assuming *C* ≃ *C*(*t*/τ_1_) and equate the limits *t*/τ_1_ → *∞* with *t*/τ_2_ → 0, yielding
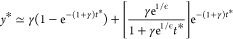
26

Type *b* dynamics with γ < 1 is characterized by the fact that the second term in eq 19 is important when y* ∼ 1 > γ. This implies that an additional regime spans from the logarithmic decay to the terminal exponential decay for 1 > y* > γ. The simplicity of our model allows us to calculate the solution of eq 19 exactly, i.e.
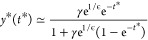
27Obviously, the logarithmic decay previously derived from eq 21 is recovered here in the limit t* ≪ 1. It is interesting to note that this second intermediate regime, when it exists, is also universal. Therefore, the break from universality is produced through the transition to the terminal exponential regime.

**Figure 11:**
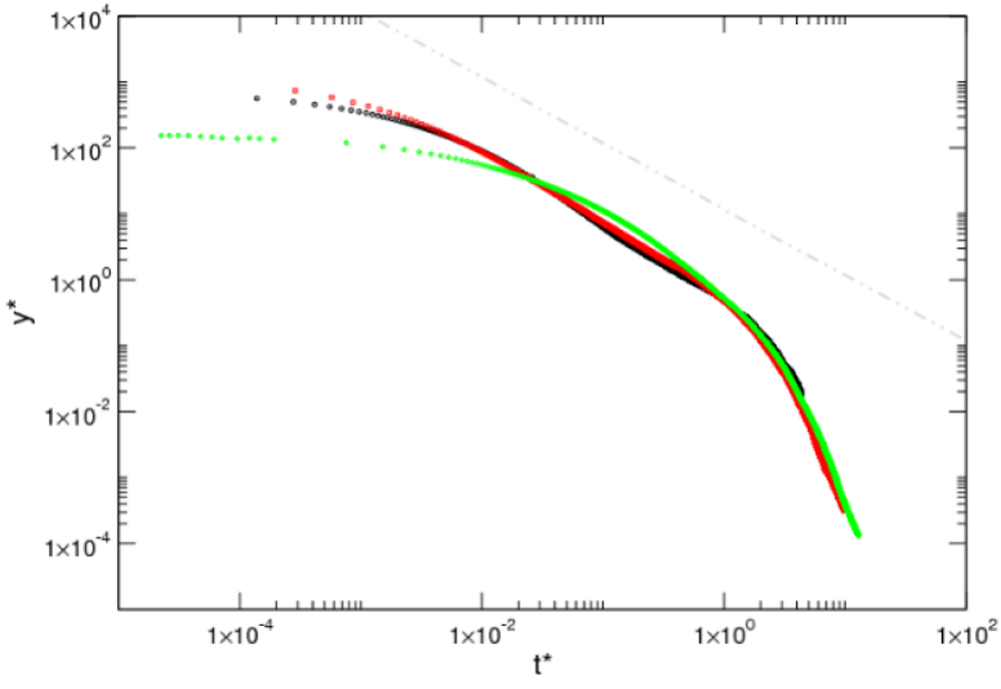
Simulation data represented with the scaling form in a log–log plot for each case study: flexible in black, semiflexible in red, and rigid in green. An additional gray dot-dashed line of slope 1 indicative of a logarithmic decay is included above the data sets.

The matching of the outer and inner solutions requires that eq 24 be rewritten as
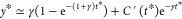
28where compared to the previous case, . Thus, in a similar way to the one needed to reach eq 26, we obtain
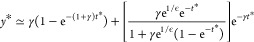
29The analysis of these asymptotic behaviors in comparison with the simulated and experimental data give rise to the following conjectures. First, the simulation results seem to lie far from the terminal exponential regime, and the deviation from the logarithmic behavior shows instead the transition toward the second intermediate regime. Because of the fact that γ is virtually negligible for the three cases, and in view of eq 29, we can consider that the terminal regime is virtually unattainable and that the observable terminal regime in Figure 11 is the second intermediate regime, which also behaves exponentially for t* ∼ 1. Effectively, from eq 29
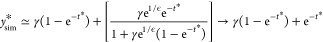
30Second, all the experimental data corresponding to γ > 1 seem to lie entirely inside the logarithmic region. Therefore, our estimate of the kinetic constant *k*_2_ for these sets is not reliable as there is a lack of data in the required nonlogarithmic region. Finally (see Figure 10), three sets display deviations from the logarithmic decay at around t* ∼ 1 (system 1, set 2; systems 1 and 2, set 3) . These three cases correspond to γ < 1, and hence we can conclude that they show the onset of the second intermediate regime and that the estimate of *k*_2_ is meaningful.

In view of these observations, we can conjecture that the experimental situations where we found γ > 1 were due to the lack of significant data outside the logarithmic decay. If we admit that the only relevant situation is thus γ < 1, then, the exponential decays detected are due to the asymptotic behavior of the second intermediate regime. Thus, the experimental curves as well as the simulation data could be well fitted with a mathematical expression of the form of eq 30, which in ordinary variables reads
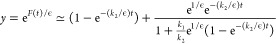
31Furthermore (see Figure 11), we observe that both the flexible and semiflexible chains show a rather wide logarithmic behavior of about 2 decades. Moreover, their behavior in scaled variables is rather coincident, enforcing our claim that the observed decay is universal, provided the polymers have a similar physical nature. Interestingly, the rigid chain does not show any trace of a logarithmic decay. In fact, no scaling of the data was found that was able to give the same master curve decay shown by the first two cases. This fact strongly suggests that the mechanism leading to the intermediate regimes is absent if the chain lacks sufficient conformational degrees of freedom. Therefore, we can also conjecture that the internal chain flexibility and the number of Kuhn segments are the key elements to understand the nature of the exotic intermediate regimes and that the observed universal behavior is based on its entropic nature.

## Conclusions

In this paper, we have analyzed the relaxation kinetics of copolymeric micellar systems at short and intermediate times. We have performed simulations of Pluronic surfactants in water at 37 °C as a function of the chain flexibility. The L44 triblock copolymer was chosen and given three different degrees of flexibility, two of which are hypothetical and do not correspond to any molecule. The surfactants were described by using a coarse-grain model in an implicit solvent of water which correctly reproduces the CMC. The dynamic simulations start with an equilibrated micelle in which the surfactants are labeled, after which we follow the kinetics of the exchange of labeled single surfactants between the micelle and the bulk.

On plotting the corresponding correlation function, we observed three well-defined regimes. In the first regime at very short times the surfactants are expelled without conformational changes, while the third regime seems to correspond to a crossover toward the terminal Halperin and Alexander exponential decay, which must exist well beyond the limit of our simulation data. The second regime only appears for the two case studies with higher chain flexibility and not for the rigid case. This intermediate regime corresponds to a characteristic logarithmic relaxation. In our previous works we used our simulation data to justify that this particular logarithmic decay arises from a degeneracy of energy states of the hydrophobic block in the micelle core, which becomes broken on entering the corona. Here, we provide qualitative proof that this process could control the initial stages but that the explanation for longer times (the intermediate regime) requires the existence of a preferred conformation—hairpin for the triblock copolymers used in this study—on exit. Such a preferred conformation is characterized by a rather crumpled hydrophobic core, which gives the lowest energy barrier to escape across the hydrophilic corona. Thus, after the initial stages of the process, chains have to diffuse in conformational space to reach such a preferred hairpin conformation on exit. The different conformations are separated by entropic barriers that should be overcome before exiting, which induces the variety of free energy barrier heights responsible for the nonexponential decay rate. It is consistent with this interpretation that a sufficient number of degenerate conformational states needs to occur in order for this second regime to appear. This requires a minimum number of Kuhn segments in the hydrophobic block. In our model six Kuhn segments were enough, but two were insufficient.

This second regime with a logarithmic relaxation has also been seen in several experimental studies using TR-SANS, and it is the interpretation of the cause of this second logarithmic regime that this article is particularly concerned with. In some experimental works this peculiar logarithmic relaxation is attributed to the polydispersity of the block copolymer samples. The justification is that the escape of chains is governed by energetic barriers in the corona, whose heights vary depending on the chain length. However, in one of our previous works we indicated that the logarithmic behavior required a rather particular form of polydispersity.^29^ Here we show that such a logarithmic behavior is rather universal for many different systems and that it is also present in our monodisperse simulations. Therefore, as we also argued in this last reference, it should be rooted in the physical nature of the system. The argument based on the polydispersity gained further weight due to the fact that in some monodisperse systems, namely poly(ethylene oxide) polymers (PEO) with *n*-alkyl ethers, the logarithmic behavior was not identified. It should be noted, however, that in these cases the hydrophobic block was limited to at most *n* = 30 monomers, which is about four Kuhn segments. In the simulation studies, six Kuhn segments were sufficient for the logarithmic regime to appear; however, two segments were not enough. Therefore, this suggests that the chains used in these works did not have the required flexibility for displaying the discussed mechanism leading to the nonexponential intermediate regime. Consequently, our conclusion is that the hydrophobic blocks of the copolymers used in the experiments are not sufficiently long for the required degeneracy of the energy states to appear. Polydispersity is doubtlessly an important factor in the chain kinetics, but our simulations indicate that the logarithmic regime can already be obtained without it if the hydrophobic block of the polymer can sample a sufficient number of degenerate configurational states in the micelle core.

Another important element in the understanding of surfactant dynamics is with regards to any conformational changes on leaving the core. As in our previous studies, although we do find a slight collapse of the hydrophobic block and a certain folding of the chain, this is a subtle effect and not a complete collapse or a full stretching of the chain as sometimes assumed in other works.

The proposed modified version of Eyring’s equation has allowed us to fit both our data and the experimental ones available in the literature onto a general master curve. An analysis of the experimental data reveals that they collapse onto a master curve in which only the logarithmic decay and the onset of the crossover toward the next regime are observed. While the logarithmic decay requires one kinetic constant *k*_1_, the crossover is governed by a second constant *k*_2_, which cannot be reliably determined in the sets where the end of the logarithmic decay has not been reached. For the cases where we obtained a reliable determination of *k*_2_, this constant was significantly larger than *k*_1_. The same is observed from the fit of the simulation data to the modified Eyring’s equation. All in all, casting these varied data under this model has allowed us to propose that this crossover regime is in fact also universal. This regime is previous to the final true Halperin and Alexander exponential decay, which lies far beyond the limit of the reported and simulated data. Finally, from an asymptotic analysis, we have proposed a curve that must phenomenologically describe the short time dynamics of this type of micellar system (eq 31), which reproduces the three observable regimes of the displayed data. A question still remains to be answered regarding the interpretation of the exponential decays observed in experimental data with respect to the Halperin and Alexander model, for example, poly(ethylene oxide) polymers (PEO) with *n*-alkyl ethers where only an exponential decay is found, which we have argued may be due to the presence of too rigid polymers without the necessary configurational states. Such an exponential decay could be the second intermediate regime predicted by the modified Eyring’s equation, rather than the Halperin and Alexander relaxation,^28^ but further experimental data and analysis are required to confirm this statement.

Finally, we like to mention some of the limitations of our methodology. Although we exactly model the intrachain interactions by explicit single-chain conformations, the mean-field nature of the interchain interactions does not allow us to include the correlated behavior of several chains. For example, within SCMF theory it is not possible to model hindrances to the chain motion due to entanglements with other chains, which may play some role in the dynamic behavior of the system. However, in view of the shortness of the chains considered in this work, we expect that their influence is not significant since the distance between entanglements may in fact be larger than the chain length. A more important limitation is that the use of an implicit solvent effectively limits the application of our model to the temperature of the experimental data used to fit the interaction parameters, in this case 37 °C. However, temperature-dependent model parameters could be developed by fitting to experimental data at other temperatures.

## Supporting Information Available

Figure S1: plot of the fitting accuracy of the modified Eyring’s equation with our correlation function simulation data for (a) flexible, (b) medium flexibility, (c) and rigid surfactant chains, in different scales; Figure S2: plot of the fitting accuracy of the modified Eyring’s equation with previous experimental data (set 1) for PEP1–PEO20 in 25 mol % DMF/water mixture at ϕ = 1 at (a) 47, (b) 55, (c) 60, and (d) 65 °C using different scales; Figure S3: plot of the fitting accuracy of the modified Eyring’s equation with previous experimental data (set 2^41^) for triblock surfactants at different concentrations (a) 1 vol % PEP–PS–PEP and (b) 6 vol % PEP–PS–PE; Figure S4: plot of the fitting accuracy of the modified Eyring’s equation with previous experimental data (set 3^42^) for two PS–PEP diblock copolymers with different hydrophobic (PS) lengths (a) ⟨*N*_PS_⟩ = 255 and (b) ⟨*N*_PS_⟩ = 412 using different scales (PDF)
