# Supplementary material for: Universal Scaling for the Exit Dynamics of Block Copolymers from Micelles at Short and Long Time Scales
Source: Macromolecules. 2022 Jan 24;55(3):914–27. doi: 10.1021/acs.macromol.1c02387 (PMC8842487; doi:10.1021/acs.macromol.1c02387)
Supplement: Supplementary file 1 — ma1c02387_si_001.pdf [file ma1c02387_si_001.pdf]

Universal Scaling for the Exit Dynamics of Block Copolymers from Micelles at Short and Long Time Scales

Maria S. Pantelidou,[†] Fabián A. García Daza,[‡] Josep Bonet Avalos,[†] and Allan D. Mackie[*,†]

†*Departament d'Enginyeria Química, ETSEQ, Universitat Rovira i Virgili, Tarragona 43007, Spain*

‡*Department of Chemical Engineering, The University of Manchester, Manchester M13 9PL, United Kingdom*

E-mail: allan.mackie@urv.cat

Supporting Information Available

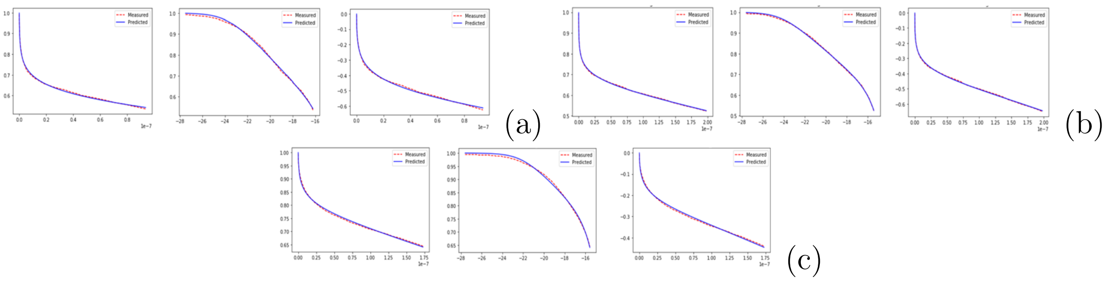

Figure S1: Fitting accuracy of the modified Eyring's equation with our correlation function simulation data for (a)*flexible*, (b) *medium flexibility*, (c) *rigid* surfactant chains, in different scales: From left to the right *Linear-Linear*, *Linear-Log* and *Log-Linear* plots.

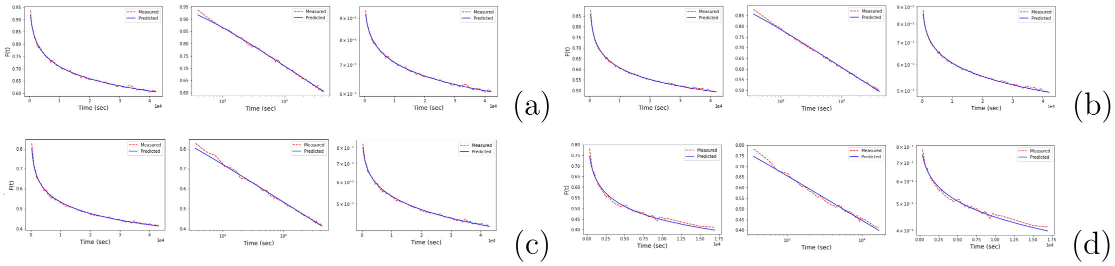

Figure S2: Fitting accuracy of the modified Eyring's equation with previous experimental data (Set 1[1]) for PEP1-PEO20 in 25 mol % DMF/water mixtrure at $\phi = 1$ at (a) 47 °C, (b) 55 °C, (c) 60 °C, (d) 65 °C using different scales. From left to the right: *Linear-Linear*, *Linear-Log* and *Log-Linear*.

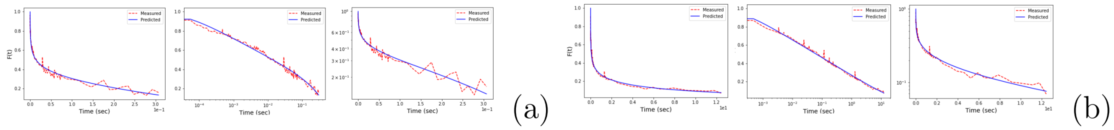

Figure S3: Fitting accuracy of the modified Eyring's equation with previous experimental data (Set 2[2]) for triblock surfactants at different concentrations (a) 1vol % PEP-PS-PEP, (b) 6vol % PEP-PS-PEP. From left to the right: *Linear-Linear*, *Linear-Log* and *Log-Linear*.

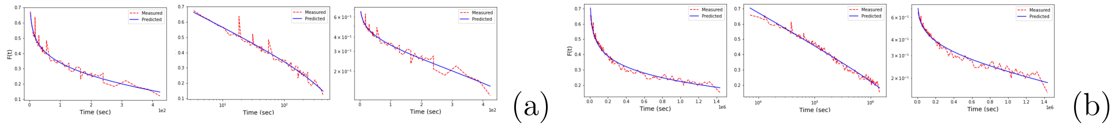

Figure S4: Fitting accuracy of the modified Eyring's equation with previous experimental data (Set 3[3]) for two PS-PEP diblock copolymers with different hydrophobic (PS) lengths (a) $\langle N_{\mathrm{PS}} \rangle = 255$ and (b) $\langle N_{\mathrm{PS}} \rangle = 412$ using different scales. From left to the right: *Linear-Linear*, *Linear-Log* and *Log-Linear*.

References

(1) Lund, R.; Willner, L.; Stellbrink, J.; Lindner, P.; Richter, D. Logarithmic chain-exchange kinetics of diblock copolymer micelles. *Physical review letters* **2006**, *96*, 068302.

(2) Lu, J.; Bates, F. S.; Lodge, T. P. Remarkable effect of molecular architecture on chain exchange in triblock copolymer micelles. *Macromolecules* **2015**, *48*, 2667–2676.

(3) Choi, S.-H.; Lodge, T. P.; Bates, F. S. Mechanism of molecular exchange in diblock copolymer micelles: hypersensitivity to core chain length. *Physical review letters* **2010**, *104*, 047802.
